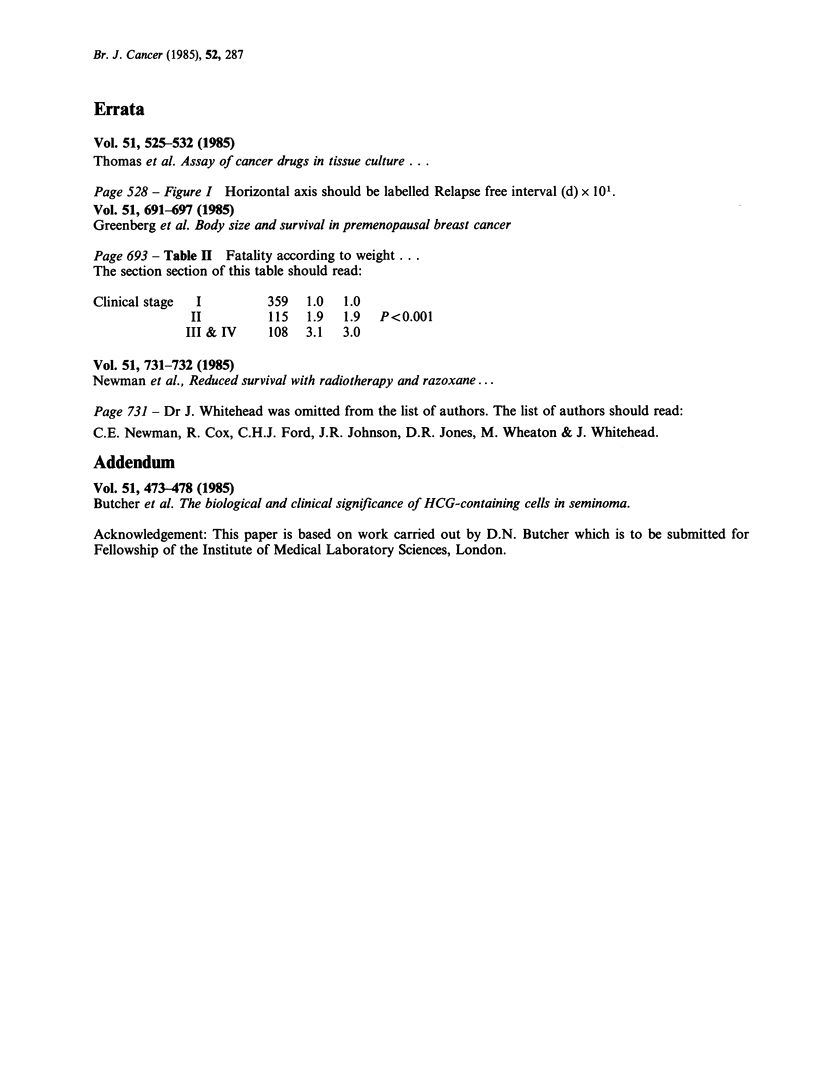# Errata

**Published:** 1985-08

**Authors:** 


### Errata

**Vol. 51, 525–532 (1985)**
Thomas *et al. Assay of cancer drugs in tissue culture . . .*

*Page 528 – Figure I* Horizontal axis should be labelled Relapse free interval (d) $\times 10^1$.

**Vol. 51, 691–697 (1985)**
Greenberg *et al. Body size and survival in premenopausal breast cancer*

*Page 693* – **Table II** Fatality according to weight . . .
The section section of this table should read:

| | | | | | |
|---|---|---|---|---|---|
| Clinical stage | I | 359 | 1.0 | 1.0 | |
| | II | 115 | 1.9 | 1.9 | $P<0.001$ |
| | III & IV | 108 | 3.1 | 3.0 | |

**Vol. 51, 731–732 (1985)**
Newman *et al., Reduced survival with radiotherapy and razoxane . . .*

*Page 731* – Dr J. Whitehead was omitted from the list of authors. The list of authors should read:

C.E. Newman, R. Cox, C.H.J. Ford, J.R. Johnson, D.R. Jones, M. Wheaton & J. Whitehead.